# Hydrophobic Polystyrene‐Modified Gelatin Enhances Fast Hemostasis and Tissue Regeneration in Traumatic Brain Injury

**DOI:** 10.1002/adhm.202300708

**Published:** 2023-07-23

**Authors:** Wenyan Li, Kaige Xu, Yuqing Liu, Xuejiao Lei, Xufang Ru, Peiwen Guo, Hua Feng, Yujie Chen, Malcolm Xing

**Affiliations:** ^1^ Department of Neurosurgery Southwest Hospital Third Military Medical University (Army Medical University) Chongqing 400038 China; ^2^ Department of Mechanical Engineering University of Manitoba 75 Chancellors Circle Winnipeg MB R3T 5V6 Canada

**Keywords:** hemostasis, neuroregeneration, platelet activation, traumatic brain injury, tuning hydrophobic chains

## Abstract

Hemostatic sealant is required to deal with blood loss, especially in the scenario of traumatic brain injury (TBI), which presents high rates of morbidity and disability. Hemostasis in surgery with traditional gelatin‐based sealants often leads to blood loss and other issues in brain because of the hydrophilic gelatin swelling. Herein, hydrophobic effects on the hemostasis in TBI surgery are studied by tuning the chain length of polystyrene (PS) onto methylacrylated gelatin (Gel‐MA). The hydrophobicity and hemostatic efficiency can be tuned by controlling the length of PS groups. The platelet activation of modified sealants Gel‐MA‐2P, Gel‐MA‐P, and Gel‐MA‐0.5P is as much as 17.5, 9.1, and 2.1 times higher than Gel‐MA in vitro. The hemostatic time of Gel‐MA‐2P, Gel‐MA‐P, and Gel‐MA‐0.5P groups is 2.0‐, 1.6‐, and 1.1‐folds faster than that in Gel‐MA group in TBI mice. Increased formation of fibrins and platelet aggregation can also be observed in vitro by scanning electron microscopy. Animal's mortality is lowered by 46%, neurologic deficiency is reduced by 1.5 times, and brain edema is attenuated by 10%. Protein expression is further investigated to exhibit toxic iron‐related processes caused by delayed hemostasis and activation of platelets via PI3K/PKC‐α signaling. The hydrophobic Gel‐MA has the potential in hemostatic TBI and promotes nervous system recovery in brain with the potentials in clinics.

## Graphical abstract

Methylacrylated gelatin sealant is modified with polystyrene to increase the hydrophobicity and efficiency in hemostasis. The implantation of polystyrene‐modified sealant in traumatic brain injury mice avoids tissue oppression and greatly enhances the hemostasis, survival, neurological performance, and neuroregeneration. Contact of polystyrene groups with platelets activates PI3K/PKC‐α signaling which triggers platelet aggregation and hemostasis.

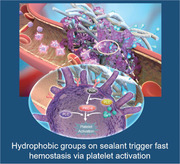

## 1 Introduction

Traumatic brain injury (TBI) happens when the head is hit by a blow and jolt, or pierced by objects. It might lead to severe bleeding and brain tissue damage, imposing great threats to human life and living quality. The incidence of TBI is about 50 000 000 per year worldwide.^[^ ^1^ ^]^ There are still more than 3 million patients suffering from different kinds of complications including neurologic deficits and psychologic issues. The clinical treatment, caretaking management and psychologic pressure lead to robust burden on government, society, and family.^[^ ^2^ ^]^

TBI causes primary and secondary damages. The primary damage is the result of instantaneous impact, brain tissue disruption, and hematoma oppression. Secondary damage stems from disordered microenvironment and hematoma degradation which will induce inflammation, iron overload, and free radical accumulation. Therefore, rapid hemostasis during operation is important for alleviating TBI secondary damages and promoting neural protection for the long term.^[^ ^3^ ^]^

Versatile types of materials have been investigated as hemostatic agents such as chitosan, cellulose, fibrins, and gelatin.^[^ ^4^, ^5^, ^6^ ^]^ Chitosan is able to activate platelet following the interaction and thus leads to thrombosis.^[^ ^7^ ^]^ Cellulose enhances platelet accumulation by its carboxyl groups.^[^ ^5^ ^]^ Materials containing fibrins facilitate robust fibrin clots in hemostasis.^[^ ^6^ ^]^ However, these polymers are disadvantageous in tissue adhesion, artificial clots construction, and excessive expansion.^[^ ^8^, ^9^ ^]^ Gelatin‐based agents which are commercially available exhibit better performance, for example, well‐proven biocompatibility, degradability, low immunogenicity, suture‐free flexibility, and porosity which absorbs blood cells.^[^ ^10^ ^]^ These features yield gelatin broad applications from bench to bedside, especially as hemostatic sealants in neurosurgery. However, for severe bleeding and hematoma formation in TBI, limited efficacy becomes an apparent defect of gelatin.^[^ ^11^ ^]^ Since gelatin contains functional groups such as lysine groups, people have been attempting to make modification to enhance its effect of hemostasis. For example, polycaprolactone (PCL) has been utilized to strengthen the mechanical and porous property of gelatin, yielding improved hemostasis.^[^ ^12^ ^]^ Another study fabricates gelatin microspheres to increase the contact surface for hemostasis.^[^ ^13^ ^]^ However, compression to adjacent tissue because of the expansion following liquid absorption^[^ ^14^ ^]^ is still another unavoidable drawback of gelatin.

To avoid excessive swelling of gelatin, hydrophobic groups are incorporated to fabricate hydrophobic groups‐conjugated gelatin sealant. In addition to reduction of water absorption, hydrophobic materials are supposed to trigger the hemostasis by enhancement of blood cell anchoring.^[^ ^15^ ^]^ For example, an alkyl chain modified catechol compound which is hydrophobic facilitates fast hemostasis of the contton gauze.^[^ ^16^ ^]^ Carbon nanofibers coated surface promotes fast clotting, reduces blood loss and withstands hemodynamic fluctuation.^[^ ^17^ ^]^ Our group also introduced a hydrophobic glue for hemostasis by its strong bonding and water repelling.^[^ ^8^ ^]^ Besides the physical hemostatic effects of hydrophobic groups, bioactive polystyrene (PS) has been explored in platelet activation since it mediates the surface activation via surface receptors.^[^ ^18^ ^]^ To be specific, glycoprotein (GP) IIb/IIIa complex on platelet surface is suggested to conduct the signals upon interaction. Level of phosphoinositide 3‐kinase (PI3K) will then be elevated, and it leads to the upregulation of protein kinase c‐α (PKC‐α), which further leads to activation of platelet synergistically with calcium and diacylglycerol (DAG).^[^ ^19^ ^]^

**Scheme 1:**
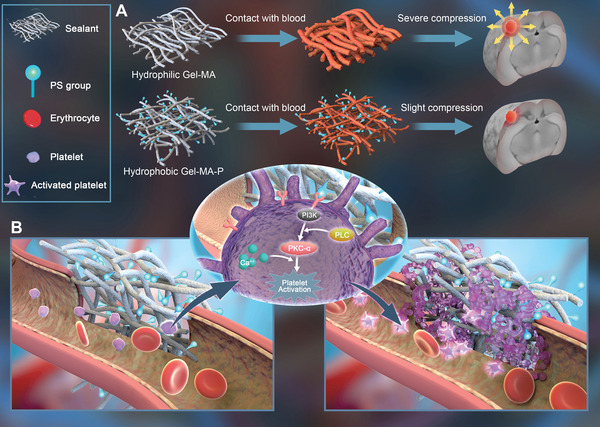
Application of sealants containing PS groups for hemostasis. A) Sealants without PS groups give rise to compression force due to the material swelling after contact with blood. Hydrophobic sealants with PS prevent the materials from expanding. B) In TBI lesion site, the implantation of PS‐abundant sealant leads to fast hemostasis by platelet activation via contact with PS.

Based on these facts, we fabricated spongy sealants of gelatin conjugated with PS in different ratios to be applied on TBI mouse model. The sealants were lyophilized before use to facilitate its self‐molding in different lesion sites. It was hypothesized that PS groups imposed hemostatic effects via platelet activation by triggering GP IIb/IIIa‐related pathway and thus reduced iron‐elicited apoptosis for protecting neuronal cells (**Scheme** **1**). As a result of the alleviated cell loss, animal survival, tissue regeneration, and functional recovery would be eventually improved. This study applied PS on gelatin as the hemostatic sealant in TBI, providing a less‐compressing, low‐cost, and fast‐activating hemostatic product. It would be of great importance for treating TBI patients to enhance their survival and neurologic functions in the future.

## 2 Results and Discussion

### 2.1 Synthesis and Characterization of Gel‐MA‐P

**Figure 1:**
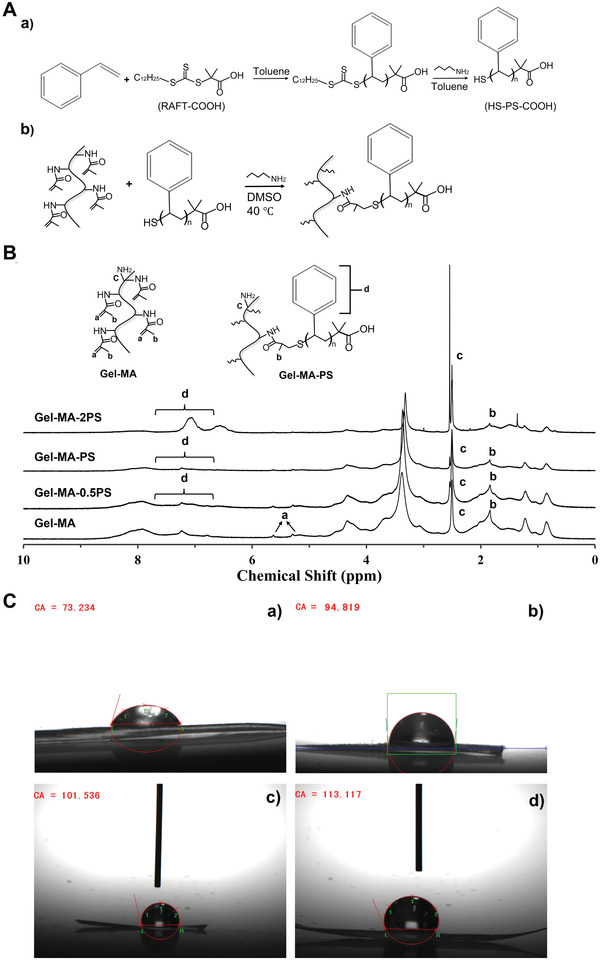
Synthesis and characterization of Gel‐MA, Gel‐MA‐0.5P, Gel‐MA‐P, and Gel‐MA‐2P. A) Synthetic route of a) PS‐SH and b) Gel‐MA‐Ps. B) ^1^H NMR spectrum of the Gel‐MA, Gel‐MA‐0.5P, Gel‐MA‐P, and Gel‐MA‐2P, respectively. C) Water contact angle tests demonstrated that hydrophobic transition after PS was conjugated on Gel‐MA, contact angles of a) Gel‐MA, b) Gel‐MA‐0.5P, c) Gel‐MA‐P, and d) Gel‐MA‐2P.

The synthetic procedure of Gel‐MA‐P with different hydrophobicity is shown in **Figure** **1**A. Briefly, PS with terminal thiocarbonylthio group via the reversible addition‐fragmentation chain transfer (RAFT) polymerization technique was synthesized using S‐dodecyl‐S′‐(α, α′‐dimethyl‐α″‐acetic acid) trithiocarbonate (RAFT‐COOH) as chain transfer agent and then transformed to α‐thiol, ω‐carboxyl telechelic polystyrene (HS‐PS‐COOH) via aminolysis with 1‐butylamine [Figure 1A(a)].^[^ ^20^ ^]^ Subsequently, the preprepared methylacrylated gelatin (Gel‐MA) was conjugated with the α‐thiol group to obtain the final products of Gel‐MA‐Ps [Figure 1A(b)]. Then, the HS‐PS‐COOH and Gel‐MA with various proportions were prepared in the same manner and recorded as Gel‐MA‐*x*P (*x* is the mass ratio of PS/Gel‐MA, and *x* is taken as 0.5, 1, and 2, respectively). Based on the ^1^H NMR spectrum of the PS (Figure S1, Supporting Information), about 89.4% of styrene was converted into PS; also, related to the weight ratio of target molecular weight of PS/RAFT−COOH = 4000/364, the final molecular weight of the PS is 3576 g mol^−1^.

The ^1^H NMR spectroscopy was adopted to verify the chemical structure. As shown in Figure 1B, in the ^1^H NMR spectrum of Gel‐MA, the peak at 5.28 and 5.62 ppm corresponding to acrylic protons of methacryloyl groups grafted on gelatin; the peaks between 1.5 and 2 ppm were assigned as methyl protons of methacryloyl groups.^[^ ^21^, ^22^ ^]^ The peaks between 2.5 and 3 ppm were due to the lysine methylene from gelatin.^[^ ^23^ ^]^ Furthermore, the spectrums of Gel‐MA‐0.5P, Gel‐MA‐P, and Gel‐MA‐2P display the new proton signals between 6.25 and 7.22 corresponding to phenyl groups from the PS.^[^ ^24^ ^]^ However, these peaks were also shown on Gel‐MA, which is because of the presence of the aromatic ring on gelatin.^[^ ^25^ ^]^

In the Fourier transform infrared (FTIR) spectra of Gel‐MA‐2P (Figure S2, Supporting Information), the absorption bands at 1632 and 1533 cm^−1^ are corresponding to amide I's C═O stretching and amide II's N─H bending vibration, respectively; and the C─N stretching vibration is at 1225 cm^−1^.^[^ ^26^, ^27^ ^]^ There is no sharp and obvious styrene vinyl group's C═C stretching at 1682 cm^−1^, which may prove that a successful polymerization of PS.^[^ ^28^ ^]^ However, this peak is affected by C═O stretching vibration (amide I) at 1632 cm^−1^ of gelatin and Gel‐MA; thus, the ^1^H NMR has been performed for further evidence (Figure S1, Supporting Information, and Figure 1B). In addition, the phenol group of O─H stretching at about 3500 cm^−1^ on PS also overrides amine N─H stretching on gelatin and Gel‐MA.^[^ ^29^ ^]^

In order to investigate hydrophobicity transition after PS conjugation on Gel‐MA, water contact angles of Gel‐MA, Gel‐MA‐0.5P, Gel‐MA‐P, and Gel‐MA‐2P were obtained and shown in Figure 1C. The Gel‐MA showed relatively high wettability with a contact angle of 73.23° [Figure 1C(a)]. However, it revealed much more water repelled after PS was conjugated as the contact angle increased to 94.82° [Figure 1C(b)]. As the amount of PS increased in conjugating with Gel‐MA, the contact angle increased to 101.54° and 113.12° for PS and 2PS, respectively [Figure 1C(c),(d)]. Thus, PS raised the Gel‐MA's hydrophobicity.

### 2.2 Hydrophobicity Triggering the Activation of Platelets and Fast Hemostasis

**Figure 2:**
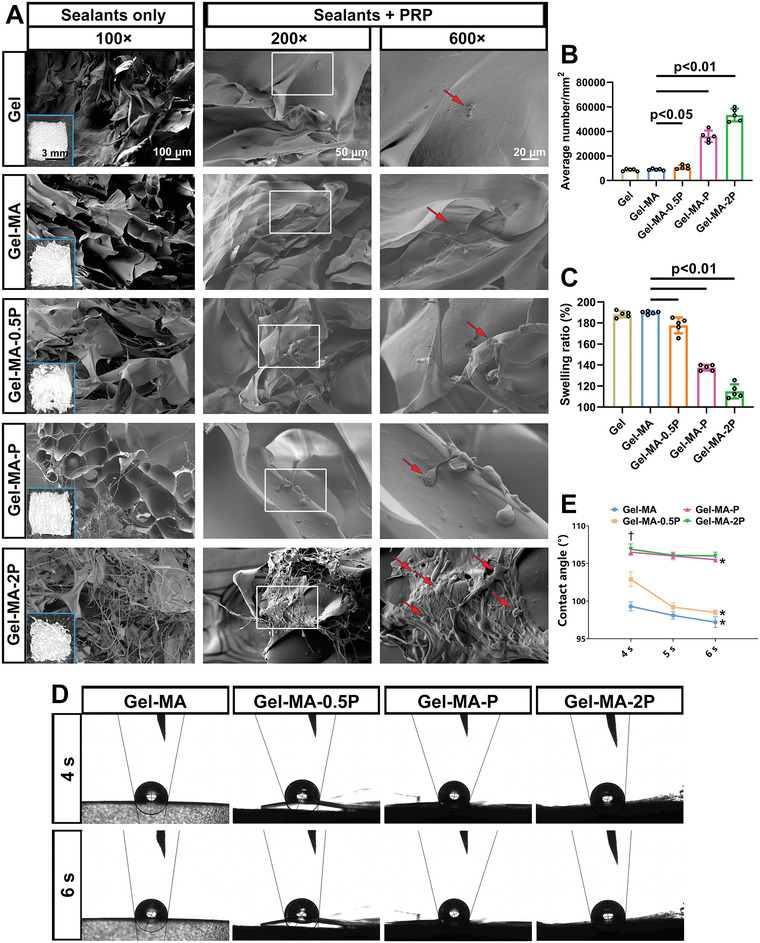
SEM of sealants and dynamic contact angle of PRP on sealants. A) Gross views of different sealants were shown in blue inserts in the left column. Images in the left column were sealants observed with SEM. In the middle column, PRP was incubated with sealants at 37 °C for 1 h and then observed with SEM. Magnified images of white‐framed inserts were exhibited in the right column. Red arrows indicate platelets. B) PRP was dropped on Gel, Gel‐MA, Gel‐MA‐0.5P, Gel‐MA‐P, and Gel‐MA‐2P to count the average number of platelets adhering to sealants (*n* = 5). C) Swelling rate of different sealants at 37 °C (*n* = 5). D) PRP was dropped on Gel‐MA, Gel‐MA‐0.5P, Gel‐MA‐P, and Gel‐MA‐2P to observe the dynamic contact angles. Angles on fourth and sixth second after contact were recorded and analyzed in (E) (*n* = 3).

Scanning electron microscopy (SEM) was applied to exhibit the interaction between platelets and sealants. Unmodified sealants were first checked under the SEM, revealing more filaments with the increase of PS proportion (**Figure** **2**A). Followed by incubation with platelet‐rich plasma (PRP) at 37 °C, Gel‐MA, Gel‐MA‐0.5P, Gel‐MA‐P, Gel‐MA‐2P, and a commercially available gelatin spongy (Gel) were observed for platelet status. As shown in Figure 2A, platelets on Gel and Gel‐MA still retained the original morphology of rounded shape. With the increase of PS chains, platelets became activated to a higher degree, exhibiting astral shape and distinct formation of clots. Especially in Gel‐MA‐2P group, coagulation was clearly triggered since plenty of platelets were shown to be bound with fibrin on the sealant. Without PS group, there were less platelets on Gel‐MA. Contrarily, stronger adhesion of platelets and more fibrin precipitation were induced when more PS groups existed, which was tremendously demonstrated in Gel‐MA‐2P. Figure 2B showed that average number of platelets adhering to sealants increased with more PS groups incorporated. He et al. reported that hydrophobic alkyl chains inhibit the blood from wicking in cotton sealants and then assist the formation of blood clots.^[^ ^16^ ^]^ Similarly, our results displayed the phenomenon that hemostasis on PS‐rich sealants was greatly activated since platelets were accumulated with fibrins adhering to the materials, indicating the interaction of hydrophobic groups with platelets which would be explored in latter parts. The rapid formation of platelet coagulation laid the basis of application in hemostasis. Meanwhile, since hydrophobic chains might change the absorption capacity of gelatin, swelling ratio of sealants calculated from water absorption rate was also examined. As calculated, swelling ratio of Gel, Gel‐MA, Gel‐MA‐0.5P, Gel‐MA‐P, and Gel‐MA‐2P was 188.26 ± 3.10%, 190.37 ± 2.62%, 176.33 ± 6.22%, 140.63 ± 4.76%, and 115.37 ± 7.09% (Figure 2C). Meanwhile, Movie S1 (Supporting Information) demonstrates the swelling status of Gel, Gel‐MA, Gel‐MA‐0.5P, Gel‐MA‐P, and Gel‐MA‐2P, with more distinct expansion in Gel and Gel‐MA than in other sealants. The incorporation of PS effectively decreased the swelling rate of sealants, potentially avoiding the oppression on adjacent tissues by the expanded sealants. In addition, since there was no significant discrepancy in basic properties between Gel‐MA and Gel, Gel‐MA was used as a control in the experiments afterward, which has also been applied in our previous research.^[^ ^6^ ^]^

The droplet of PRP was pipetted on the surface of sealant from Gel‐MA, Gel‐MA‐0.5P, Gel‐MA‐P, and Gel‐MA‐2P groups. The dynamic contact angle of the PRP with sealant surface was recorded with a camera and calculated. As time passed, PRP droplets tended to immerse within the sealants, and they immersed faster in the Gel‐MA group than in other groups (Movie S2, Supporting Information). Figure 2D showed the views of contact angle on the fourth and sixth second after droplet contact. To be specific, contact angles of Gel‐MA at 4 and 6 s were 99.30 ± 0.60° and 97.20 ± 0.70°; those of Gel‐MA‐0.5P were 102.90 ± 1.00° and 98.50 ± 0.30°; those of Gel‐MA‐P were 106.50 ± 0.40° and 105.50 ± 0.30°; those of Gel‐MA‐2P were 106.90 ± 0.70° and 106.00 ± 0.50°. When comparing the change of angles at 4 and 6 s within each sealant group, it could be found that droplet on Gel‐MA‐2P maintained a relatively steady contacting morphology (no significant difference detected for contact angle at 4 and 6 s) but immersed dramatically in Gel‐MA, Gel‐MA‐0.5P, and Gel‐MA‐P groups (contact angle comparison of 4 s vs 6 s: *p* = 0.017, 0.002, and 0.026 separately). Among different sealants on 4 s, PRP on Gel‐MA‐2P and Gel‐MA‐P illustrated larger contact angles than that on Gel‐MA and Gel‐MA‐0.5P groups, indicating the remarkable hydrophobicity in PS‐rich groups (Figure 2E). Contact angle test reflects the hydrophobicity of the substrate surface. The angle becomes larger with the increasing of hydrophobicity. Since the surface of sealants with PS groups was more hydrophobic, the PRP drop tended to display larger contact angles on these groups than on Gel‐MA group. Interestingly, via the dynamic contact angle recording, PRP droplets immersed faster into sealants with more PS groups. This may be explained that activated platelets clots and precipitated fibrins prevented the rapid immersion of PRP drops, since hydrophobic groups have been found to activate platelets to enhance the their adhesion for effective coagulation.^[^ ^30^ ^]^

**Figure 3:**
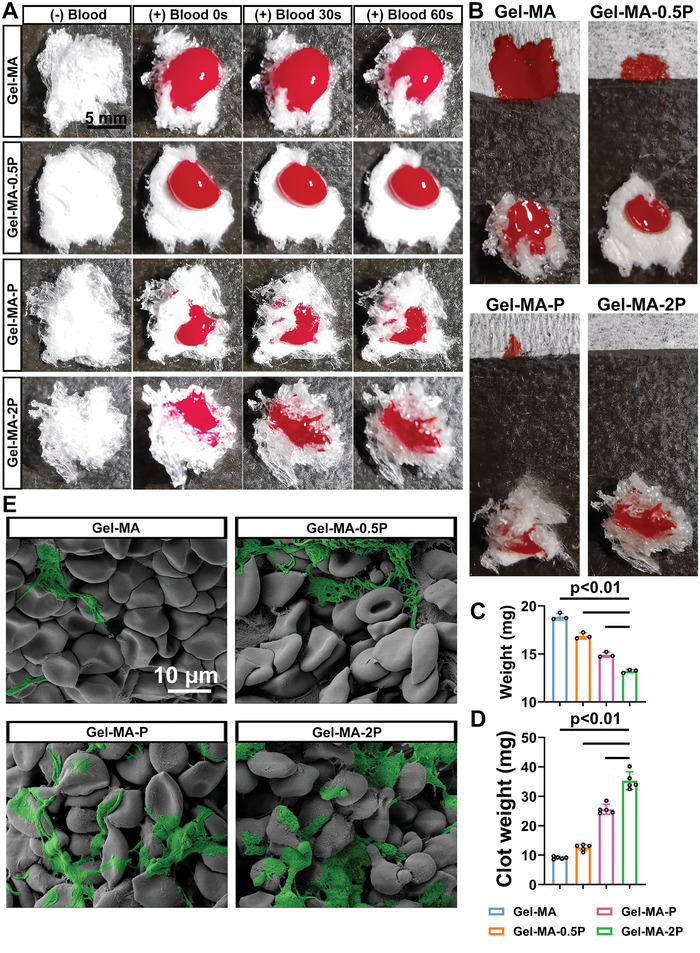
Hemostatic effect in vitro. A) Whole blood of 10 µL was dropped on 3 mg of sealants from each group, and the hemostasis could be directly observed with time passing. B) 60 s after the contact of sealants with the blood drop, a filter paper (cut in 20 mm × 20 mm, 13 mg) was used to tip on the bloodstain for demonstrating the unclotted blood. C) The tipped filter paper in different groups was weighed (*n* = 3). D) Blood clot formed in different groups was weighed (*n* = 5). E) These sealants with blood were examined with SEM. Pseudocolor (green) indicated the formed fibrins.

Hemostatic effect of different sealants in vitro is shown in **Figure** **3**. Whole blood was dropped onto the sealants to monitor the dynamic of hemostasis (Figure 3A). After 60 s, a filter paper was used to tip on the blood drop and weighed. From the gross view in Figure 3B and weight in Figure 3C, hemostasis was much more distinct in Gel‐MA‐2P group. Weight of blood clot from different groups was obtained, showing that more clot was formed with PS group increasing (Figure 3D). SEM on sealants illustrated that there would be more fibrins formed when more PS groups were coupled with, indicating enhanced hemostatic efficiency in these groups (Figure 3E).

### 2.3 Sealants with High Hydrophobic Chains Leading to Faster Hemostasis in TBI Mice

**Figure 4:**
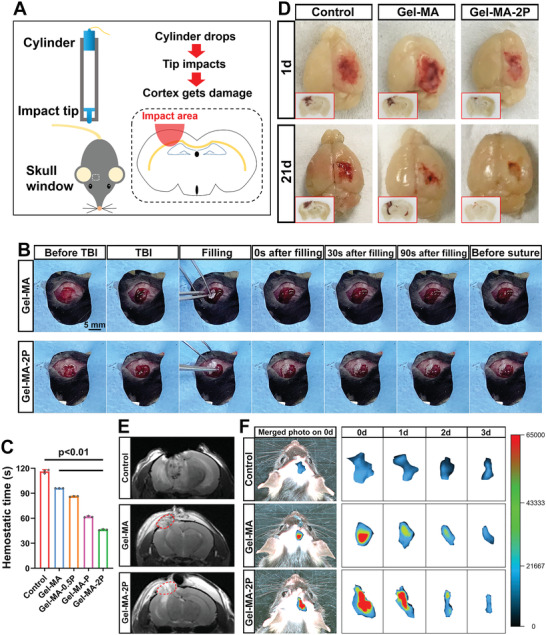
Application of sealants on TBI mouse model and the hemostatic effect. A) Schemes of the cortical impact device, skull window of mouse, and the injury mechanism. B) Procedures of sealant implantation Gel‐MA and Gel‐MA‐2P groups in TBI mice. Views of the surgical fields at different timepoints such as “before TBI,” TBI onset, sealant inserting, 0 s/30 s/90 s post insertion and “before suture” were demonstrated. C) Hemostatic time during operations without sealants and with Gel‐MA, Gel‐MA‐0.5P, Gel‐MA‐P, and Gel‐MA‐2P was counted and analyzed (*n* = 3). D) Gross views and coronal sections (red inserts) of brains in control, Gel‐MA, and Gel‐MA‐2P groups for showing hemorrhage. E) Cranial MRI of mice in control, Gel‐MA, and Gel‐MA‐2P groups. Red circles indicated inserted sealants. F) In vivo fluorescence imaging of mice in control, Gel‐MA, and Gel‐MA‐2P groups. Sealants labeled with AF488 were used in these animals. Images were taken from 0 to 3 d, and the fluorescent regions were snapped.

Since these blood‐contacting sealants are foreign, the hemocompatibility tests are important to indicate their safety. Hemolysis test was performed on Gel, Gel‐MA, Gel‐MA‐0.5P, Gel‐MA‐P, and Gel‐MA‐2P, showing the ratios below 1% (Figure S3A, Supporting Information) which is acceptable extensively.^[^ ^31^ ^]^ Hemostatic effect was verified in vivo. Animals were categorized into sham, control, and treatment groups. To ensure the biocompatibility of sealants, lactate dehydrogenase (LDH) release assay was performed prior to the surgery. Neural stem cells were cultured in a 24‐well plate with Gel‐MA, Gel‐MA‐0.5P, Gel‐MA‐P, and Gel‐MA‐2P, and the LDH release assay on 24 h indicated the biocompatibility of the sealants (Figure S3A, Supporting Information). Cortical impact device and the procedure of TBI model establishment are depicted in **Figure** **4**A. The stereotaxic instrument with the impact device is shown in Figure S4A (Supporting Information). In the surgical area, skull window for positioning the impact tip was opened with an electric drill (Figure S4B,C, Supporting Information). Surgeries applying Gel‐MA and Gel‐MA‐2P are shown in Movie S3 (Supporting Information) to demonstrate the process of hemostasis, which could be illustrated in Figure 4B at the same time. Fresh blood could be seen in lesion sites in both groups, which mimicked the realistic bleeding in TBI. After filling sealants for 30 s, bleeding in Gel‐MA‐2P stopped and the remained sealant stayed in solid form. On the contrary, Gel‐MA sealant was rapidly immersed by blood even upon contact, so that the bleeding continued even before suture. Obviously, sealant Gel‐MA‐2P contributed to faster hemostasis than Gel‐MA did (Figure 4C). During surgery, coagulation time was recorded as 118.67 ± 3.76, 96.68 ± 2.03, 85.31 ± 1.20, 62.34 ± 1.86, and 47.66 ± 4.98 s in control, Gel‐MA, Gel‐MA‐0.5P, Gel‐MA‐P, and Gel‐MA‐2P groups, demonstrating significantly shortened hemostatic time in Gel‐MA‐P and Gel‐MA‐2P groups. Afterwards, brains of mice at the time points 1 and 21 d were removed to show the appearance of the lesion and were further cryosectioned to demonstrate the coronal plane of lesion sites. Bloodstain in Gel‐MA‐2P group was much fewer than in control and Gel‐MA groups, proving the hemostatic effect of the hydrophobic material (Figure 4D). Magnetic resonance imaging (MRI) indicated the sealants in the lesion sites on 1 d (Figure 4E). For observing the degradation of sealants, Gel‐MA and Gel‐MA‐2P labeled with AF488 were applied. In vivo imaging suggested that sealants could be degraded within 3 d (Figure 4F).

Intracranial hematoma accompanied with TBI imposes great threat to human life and neurologic functions. Currently, there are several types of hemostatic devices applied in TBI surgery. The most widely used is the gelatin sponge. This device is highly absorbent as exhibited in Figure 2C so that they facilitate the hemostasis under situations where ligation is impractical. It is sometimes placed subdurally over cerebral tissue to prevent postoperative surgical adhesions.^[^ ^32^ ^]^ However, hemostasis time of gelatin sponge takes longer as shown in Figure 4B,C. Also, it was pointed that epidural fibrosis developed more often with the use of gelatin sponge.^[^ ^33^ ^]^ The compressing effect of swelling gelatin is still a drawback that cannot be neglected. Another commonly used device is injectable sealant, such as the fibrin sealant which functions to seal dura and aid hemostasis. Prior to injection, the sealant has to be mixed to activate fibrinogen into fibrin to produce layers of adhesion, mimicking coagulation in focal sites.^[^ ^34^ ^]^ Nonetheless, the sole injectable sealants are not applicable in controlling massive bleeding and may bring about fatal thromboembolism when entering vessels.^[^ ^35^ ^]^ Therefore, a fast‐hemostatic, less‐absorbent, and biosafe sealant is required. In this study, sealants crosslinked with PS hydrophobic chains exhibited faster hemostasis, less swelling ratio, satisfied biocompatibility, and fast degradation, conferring their availability in animals. Gel‐MA‐2P successfully decreased the bleeding from the TBI lesion, which was evidenced by Movie S3 (Supporting Information) and brain dissection (Figure 4), indicating PS hydrophobic groups incorporated sealants were ideal hemostatic devices.

### 2.4 Application of Sealants with High Hydrophobic Chains Content Yielded Enhanced Animal Survival and Functional Recovery

**Figure 5:**
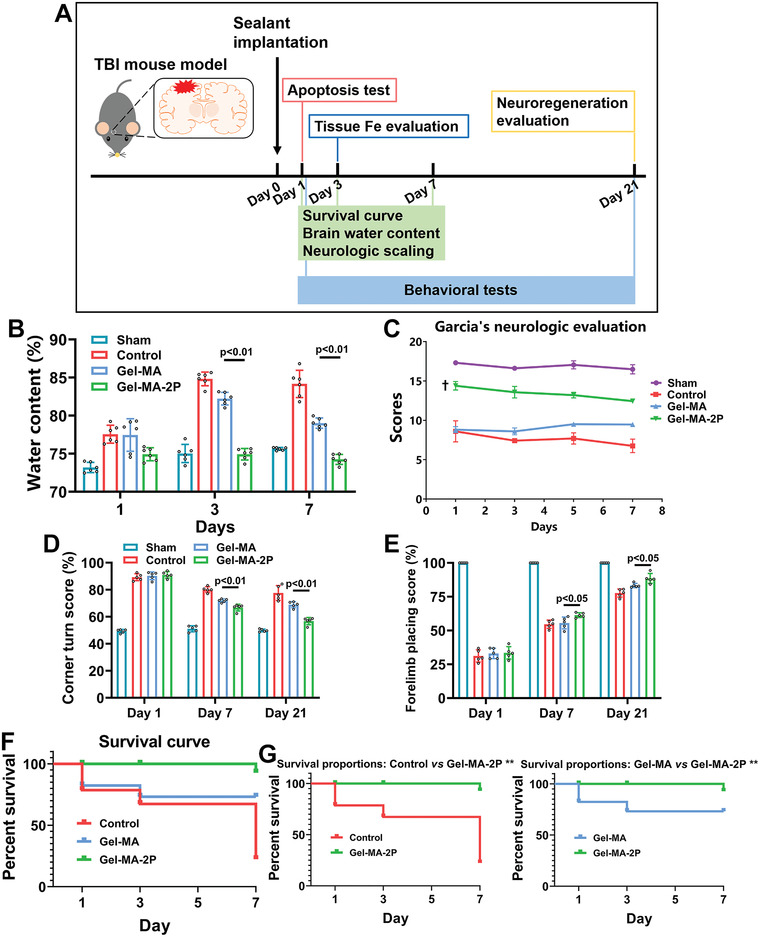
Neurological evaluation of TBI mice after sealant implantation. A) Experimental schedule of animal studies. B) Brain edema of animals from different groups at different time points was assessed by calculating the brain water content (*n* = 6). C) Garcia's score for evaluating neurologic functions of TBI mice from different groups at different time points after surgery (^†^ *p* < 0.01 when Gel‐MA‐2P vs Gel‐MA from day 1 to day 7) (*n* = 3). D,E) On different time points, corner turn and forelimb tests were performed to examine the motor functions (*n* = 5). F,G) On day 1, day 3, and day 7, survival of mice in different groups was analyzed, and survival curves were drawn.

Since the hemostatic effect of hydrophobic sealants was remarkable, the effects on animals were monitored. Experimental schedule was shown in **Figure** **5**A. Mortality rate was figured based on the number of dead mice during operation or immediately post operation. As shown in Figure S3B (Supporting Information), mortality in sham, control, Gel‐MA, Gel‐MA‐0.5P, Gel‐MA‐P, and Gel‐MA‐2P was 0.00 ± 0.00%, 53.30 ± 6.70%, 46.70 ± 6.70%, 40.00 ± 0.00%, 13.30 ± 6.70%, and 6.70 ± 6.70% (*n* = 5). It could be seen that TBI mice receiving Gel‐MA‐P and Gel‐MA‐2P exhibited much lower mortality rate than Gel‐MA did. Among the mice alive, survival condition was noted at 1, 3, and 7 d, for investigating their survival rates. Mice in sham group stayed survived from day 1 to day 7. For control, Gel‐MA, Gel‐MA‐0.5P, Gel‐MA‐P, and Gel‐MA‐2P groups, animals exhibited survival rates of 61.10 ± 20.00%, 61.10 ± 20.00%, 88.89 ± 11.10%, 100.00 ± 0.00%, 100.00 ± 0.00% on day 1; 44.40 ± 5.60%, 50.00 ± 16.70%, 88.89 ± 11.10%, 100.00 ± 0.00%, 100.00 ± 0.00% on day 3; 16.70 ± 16.70%, 50.00 ± 16.70%, 77.78 ± 11.10%, 91.70 ± 8.30%, 93.30 ± 6.70% on day 7 (Figure S3C, Supporting Information). Since brain edema is a common complication of TBI and also the cause of neurologic deficits, brain water content was examined 1, 3, and 7 d after TBI (Figure 5B). Animals in sham group showed a relatively constant water content from 73.31 ± 0.75% to 75.60 ± 0.40%. No significant difference was found among groups on 1 d. On 3 d, brain water contents of Gel‐MA and Gel‐MA‐2P were 82.55 ± 1.10% and 75.07 ± 0.65%, revealing lower water content in and Gel‐MA‐2P; while on 7 d, Gel‐MA‐2P showed the water content of 74.23 ± 0.43% which was significantly lower than 78.78 ± 0.87% in Gel‐MA. Obviously, TBI mice demonstrated high water content of brains, and the application of Gel‐MA‐2P decreased it significantly. Meanwhile, neurologic behaviors of animals were rated with Garcia's Neurologic Score, corner turn test, and forelimb placing test. For Garcia's Score, when comparing Gel‐MA‐2P with Gel‐MA on day 1, day 3, day 5, and day 7, the *p*‐values were 0.0011, 0.010, 0.0012, and 0.0002 (Figure 5C). Similarly in corner turn and forelimb placing tests, animals in Gel‐MA‐2P obtained higher scores than those in Gel‐MA (Figure 5D,E). Therefore, animals receiving Gel‐MA‐2P performed better. The survival curve was created in Figure 5F, and there were significant differences when control versus Gel‐MA‐2P and Gel‐MA versus Gel‐MA‐2P (Figure 5G).

As acknowledged, TBI leads high mortality and survivors were left with neurologic deficits. Among factors of TBI, hematoma formation is a major one which results in primary physical oppression and secondary sustained damages, and also constitutes a challenging task during surgery.^[^ ^36^ ^]^ From our results, the use of Gel‐MA‐P and Gel‐MA‐2P decreased the mortality of TBI animals during and after surgery, indicating the importance of fast hemostasis in protection against mechanical oppression and the following secondary damages. These microenvironmental conditions are closely related to the neurologic functions, which can be suggested by neurologic scaling systems. Garcia's Score and functional tests we applied provided a general status of mice functions and also the recovering procedure postoperation. Notably, animals’ conditions turned worse from day 3 after surgery, which could be explained that the stress reaction protected animals during and immediately after surgery; however, brain edema and other detrimental factors prevailed from day 3.^[^ ^37^ ^]^ With the time passing, beneficial effects of hemostasis emerged as the survival, motor function and brain edema were promoted.

### 2.5 PS‐Incorporated Sealants Prevented Apoptosis and Benefited Neuroregeneration

**Figure 6:**
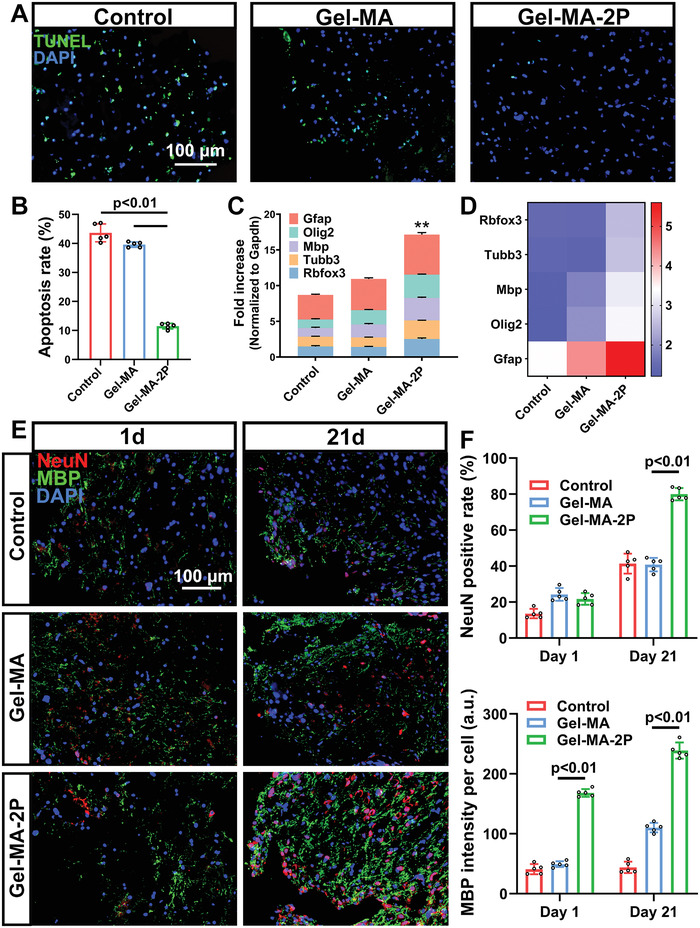
The influence on cell apoptosis and neural regeneration by different sealants after TBI. A) Brain sections of mice from control, Gel‐MA, and Gel‐MA‐2P on day 1 underwent TUNEL assay. B) Statistic of TUNEL‐positive rate of different groups based on images of five randomly captured fields in lesion sites (*n* = 5). C) Real‐time qPCR of different neuronal markers on day 21 were exhibited. The results were normalized against Gapdh expression. **p < 0.01 for the expression of Gfap, Olig2, Mbp, Tubb3, and Rbfox3 in Gel‐MA‐2P versus those in Gel‐MA (*n* = 3). The heatmap was depicted in (D). E) Brain sections of mice from control, Gel‐MA, and Gel‐MA‐2P on day 1 and day 21 underwent immunofluorescence staining, with NeuN as the neuron marker and MBP as the oligodendrocyte marker. F) Statistic of NeuN‐positive rate of different groups based on images of five randomly captured fields in lesion sites, and the MBP signal intensity per cell from these fields (*n* = 5).

Since the general influence of the applications of different sealants has been demonstrated, their impact on brain tissue was next investigated. As acknowledged, the persistent hematoma would elicit secondary damage following TBI, leading to cell apoptosis and disruption of brain tissue. Cryosectioned brain slices from mice receiving TBI and TBI + sealants on 1 and 21 d were prepared. TUNEL assay was performed on 1 d samples for observing the cell apoptosis under different circumstances of hemostasis in the acute phase. Images of five randomly captured fields adjacent lesion sites under a microscope were analyzed to calculate the TUNEL‐positive cell rates (**Figure** **6**A). From the images and the statistic, apoptotic cells per field accounted for 42.35 ± 3.24%, 39.11 ± 1.91%, and 11.71 ± 1.54% in control, Gel‐MA, and Gel‐MA‐2P groups, illustrating notably decreased apoptosis rate in Gel‐MA‐2P groups (Figure 6B).

At the same time, quantitative real‐time polymerase chain reaction (qPCR) was performed to evaluate the expression of marker genes for different cell types. On day 21, the qPCR result exhibited that all three types of neuronal cells were upregulated in Gel‐MA‐2P, compared with those in control and Gel‐MA groups (Figure 6C,D). Immunofluorescence staining for NeuN and MBP was then processed on brain slices on 1 and 21 d to show whether different hemostatic conditions affected expressions of neurons and oligodendrocytes in the acute and chronic phases (Figure 6E). Same approaches of counting and statistic were used as in TUNEL analysis (Figure 6F). It showed that NeuN positive ratio was 12.66 ± 3.51%, 24.41 ± 2.32%, and 22.64 ± 3.11% in control, Gel‐MA, and Gel‐MA‐2P on 1 d; 40.86 ± 5.40%, 42.53 ± 4.33%, and 78.44 ± 4.16% in these groups on 21 d. For MBP which is the marker of oligodendrocytes staining myelin, signal intensity was quantified and then divided by cell number per field. Therefore, the MBP intensity/cell was 39.18 ± 7.48, 53.38 ± 10.30, and 169.28 ± 9.67 in control, Gel‐MA, and Gel‐MA‐2P on 1 d; 43.33 ± 6.32, 110.42 ± 8.34, and 230.53 ± 11.95 in these groups on 21 d. Generally, there was more profound neurogenesis in Gel‐MA‐2P on 21 d. For MBP intensity, it could be drawn that the use of Gel‐MA‐2P prevented the myelin from early destruction on 1 d and further promoted the remyelination on 21 d. Therefore, the application of sealants with high content of hydrophobic chains assisted the long‐term neuronal cells regeneration.

### 2.6 PS‐Conjugated Sealants Attenuated Fe‐Related Apoptosis and Enhanced Platelet Activation via PI3K/PKC‐α

**Figure 7:**
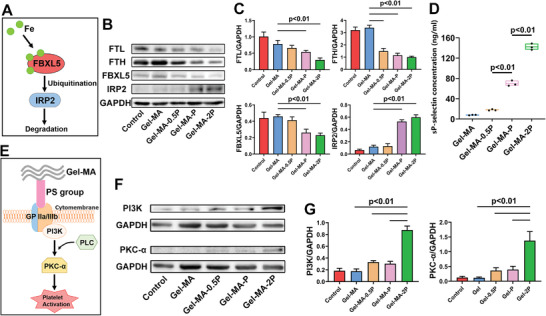
Investigation of signaling pathways associated with Fe and platelet activation. A) Scheme of the Fe‐related pathway. B) FTL, FTH, FBXL5, and IRP2 expression in tissue of lesioned brain from control, Gel‐MA, Gel‐MA‐0.5P, Gel‐MA‐P, and Gel‐MA‐2P was examined with WB. C) Statistic of WB quantification for protein level (*n* = 3). D) Evaluation of platelet activation by ELISA of sP‐selectin release in Gel‐MA, Gel‐MA‐0.5P, Gel‐MA‐P, and Gel‐MA‐2P (*n* = 3). E) Scheme of platelet activation pathway. F) WB of PI3K/PKC‐α in tissue of lesioned brain from control, Gel‐MA, Gel‐MA‐0.5P, Gel‐MA‐P, and Gel‐MA‐2P, and the quantification was obtained in (G) (*n* = 3).

Since iron is a major metabolic hazard from hematoma, Fe‐related signaling was investigated (**Figure** **7**A). Three days after animals received TBI and sealant implantation, brains were removed and protein samples in lesion part were extracted for WB (Figure 7B) and the quantification was in Figure 7C. Ferritin light chain (FTL) which consists of ferritin was found significantly reduced in Gel‐MA‐P and Gel‐MA‐2P groups, compared with that in Gel‐MA (reduced by 1.44‐ and 2.63‐folds approximately), exhibiting a hydrophobic chain‐dependent manner. Similar situation was found in ferritin heavy chain (FTH) level detection, to be specific, FTH in Gel‐MA‐0.5P, Gel‐MA‐P, and Gel‐MA‐2P was about 2.28, 2.96, and 3.40 times lower than that in Gel‐MA group. It implied that increased hydrophobic groups lessened formation of ferritins, by means of better hemostatic effect. F box and leucine‐rich repeat protein 5 (FBXL5) decreased remarkably in Gel‐MA‐P and Gel‐MA‐2P groups by around 43% and 50% when compared with that in Gel‐MA, which was as expected since FBXL5 is often viewed as an iron sensor. This protein of F‐box family will be downregulated in an iron‐limiting status and then its ubiquitination of iron‐regulatory protein 2 (IRP2) hindered. The figure also proved that IRP2 level in Gel‐MA‐P was 4.42 times of that in Gel‐MA, and in Gel‐MA‐2P was 5.08 times of that in Gel‐MA. The elevation was the result of hindered degradation by upstream FBXL5 reduction. The protein level changes of FBXL5/IRP2 axis evidently suggested that the use of sealants with hydrophobic groups helped fast hemostasis, resulting in less ferritin produced in lesion tissue and thus FBXL5 function was inhibited to give rise to IRP2 accumulation.

These results verified that sealants with PS groups sufficiently decreased iron accumulation by fast hemostasis. Importantly, iron is released from the ferritin of hemorrhage following TBI, and also comes from the degradation of red blood cell lysis. It underlies a major cause of secondary damage of neuronal tissue and cells, as well as the brain edema.^[^ ^38^ ^]^ Ferritin which consists of FTL and FTH is regarded as an indicator for iron level. As the iron increases, FBXL5 is elevated for ubiquitinating IRP2 which will be then degraded. As a result, accumulation of free iron is enhanced and causes apoptosis.^[^ ^39^ ^]^ At the same time, increased Fe level after TBI was regarded to give rise to reactive oxygen species (ROS) generation, lipid peroxidation, inflammation, and so on. These procedures robustly resulted in cell apoptosis and hindered regeneration.^[^ ^40^ ^]^ The results above demonstrated corroborated facts that TUNEL positive rate in TBI mice was tremendous and the PS‐crosslinked sealants alleviated the apoptosis. At the same time, with the efficient hemostasis in Gel‐MA‐P and Gel‐MA‐2P groups, microenvironment in focal sites was ameliorated so that neurogenesis and oligodendrogenesis were promoted. As suggested, although iron is indispensable for development and functions of nervous system, the abnormal accumulation will be hazardous, especially for neurons and oligodendrocytes.^[^ ^41^, ^42^ ^]^ As such, boosting hemostasis for elimination of excessive iron met the demand of controlling Fe level for nervous system regeneration.

As a universally applied indicator for platelet activation, level of sP‐selectin was observed following contact of plasma with sealants. PRP solutions were incubated with Gel‐MA, Gel‐MA‐0.5P, Gel‐MA‐P, and Gel‐MA‐2P, respectively, for 1 h at 37 °C and then processed ELISA of sP‐selectin. Quantification of sP‐selectin showed 8.21 ± 0.37, 17.14 ± 2.04, 74.54 ± 5.71, and 143.99 ± 7.93 ng mL^−1^ in Gel‐MA, Gel‐MA‐0.5P, Gel‐MA‐P, and Gel‐MA‐2P groups (Figure 7D). It was supposed that contact of PRP with Gel‐MA‐P and Gel‐MA‐2P was sufficient to elicit platelet activation and the extent is in direct proportion to PS amount. Furthermore, PI3K/PKC‐α which was suggested to be involved in platelet activation by hydrophobic groups were examined (Figure 7E). Level of the hallmark proteins was investigated through WB (Figure 7F). PRP was incubated with four groups of sealants for 1 h at 37 °C to extract protein samples as the treatment groups. PRP protein samples were prepared as the control group. WB analysis illustrated that PI3K protein level in Gel‐MA‐2P was 4.98, 2.65, and 2.88 times of that in Gel‐MA, Gel‐MA‐0.5P, and Gel‐MA‐P groups; and PKC‐α level in Gel‐MA‐2P was 12.55, 3.83, and 3.54 times of that in Gel‐MA, Gel‐MA‐0.5P, and Gel‐MA‐P (Figure 7G). Therefore, Gel‐MA‐2P significantly triggered the upregulation of PI3K and PKC‐α.

To date, various biomaterials have been applied in surgical hemostasis. These approaches leverage different aspects in blood coagulation, for example, vessel compression, platelet activation, and coagulation induction.^[^ ^43^, ^44^ ^]^ However, brain tissue is not supposed to tolerate mechanical compression caused by sealant swelling, so that hydrophobic materials such as PS‐conjugated sealants might be more appropriate. Gel‐MA‐P we applied in TBI surgery tremendously enhanced the activation of platelets and inhibited the compression against adjacent tissues. Activation of platelets causes contraction in morphology to strengthen the clots.^[^ ^45^ ^]^ Secretion changes of sP‐selectin in our finding reflected the platelet activation condition since it is an established marker for platelet aggregation and stabilization.^[^ ^46^ ^]^ Based on previous studies, surface receptor GPIIb/IIIa is regarded to be crucial for platelet activation, adhesion and aggregation.^[^ ^47^ ^]^ Nanomaterials have been confirmed to interact with GPIIb/IIIa to trigger the downstream signaling for platelet activation.^[^ ^48^ ^]^ We found the level of PI3K and PKC‐α was markedly upregulated in PS‐rich groups, which could be explained by existing studies. Chen et al. verified nonphysiologic factor was able to trigger the platelet activation via GPIIb/IIIa‐PI3K, instead of other glycoprotein receptors.^[^ ^49^ ^]^ Meanwhile, blockade of GPIIb/IIIa is associated with platelet‐activating factor (PAF)‐induced P‐selectin expression and PKC activity, which eventually leads to platelet incapability.^[^ ^50^ ^]^ Furthermore, during the signaling following contact of PS with GPIIb/IIIa, PI3K might elicit PKC‐α in a synergistic manner with DAG and Ca^2+^.^[^ ^51^ ^]^ Taken together, the mechanism of how the sealants with PS groups triggered hemostasis was preliminarily unveiled and would inspire further studies on materials for fast hemostasis.

## 3 Conclusion

In summary, we have designed a novel hemostatic sealant with PS chains on gelatin for TBI surgery. This sealant possessed outstanding properties such as nonexpansion, economy and rapid hemostasis. When contacting with gelatin‐PS sealants, platelets were shown to be activated and bound with fibrins. TBI mice model using these PS‐rich sealants exhibited faster hemostasis, improved survival, higher neurologic scores, and controlled brain edema. In brain lesion site, cell apoptosis was greatly inhibited on mice receiving high‐contented PS, with promoted neurogenesis and oligodendrogenesis in chronical stage. The cellular protection by fast hemostasis was presumably due to hindered Fe‐related signaling, and the hemostatic effect stemmed from platelet activation mediated by GP IIb/IIIa‐associated PI3K/PKC‐α pathway. This work contributed to the modification of traditionally utilized surgical sealant in TBI, which leveraged the platelet activation by hydrophobic materials and would be potentially applied for improving patients’ life quality in the near future.

## 4 Experimental Section

### Materials

Gelatin was purchased from Ward's Science. Styrene (99.5% stab. with 4‐*tert*‐butylcatechol), 1‐butylamine (99%), and methacrylic anhydride (94%, stab. with ≈0.2% 2,4‐dimethyl‐6‐*tert*‐butylphenol) were purchased from Alfa Aesar. S‐Dodecyl‐S′‐(α, α′‐dimethyl‐ α″‐acetic acid) trithiocarbonate (RAFT‐COOH) chain transfer agent was prepared based on the previous report.^[^ ^52^ ^]^ Gelatin sponge was from Jinling Pharmaceutical Company Limited (Jiangsu, China). LDH Cytotoxicity assay kit, DAPI, and TUNEL assay kit were obtained from Beyotime Biotechnology. Primary antibodies of FTL, FTH, FBXL5, MBP, and NeuN were from Abcam. Primary antibodies of PI3K and PKC‐α were obtained from Cell Signaling Technology. Primary antibody of IRP2 was obtained from Novus. Primary antibody of GAPDH was purchased from Santa Cruz. Secondary antibodies for immunofluorescence staining were purchased from Proteintech. Horseradish peroxidase (HRP)‐conjugated secondary antibodies were purchased from Beyotime Biotechnology. sP‐selectin ELISA kit was purchased from R&D Systems. C57BL/6 mice were purchased from SPF (Beijing) Biotechnology Co., Ltd. All other chemicals were purchased from Sigma‐Aldrich and used without purification except specially indicated. All procedures which involve mice were approved by Laboratory Animal Welfare and Ethics Committee of the Third Military Medical University (No. AMUWEC20192038).

### Synthesis of Gelatin Methacryloyl

Synthesis of Gel‐MA macromolecule with a methacryloyl graft was followed from a previously published literature.^[^ ^20^ ^]^ Briefly, 10% w/v gelatin was completely dissolved in deionized water at 50 °C by stirring. Then, MA (0.8 mL MA/g gelatin) was added into gelatin solution and stirred at 50 °C for another 2 h. The reaction was quenched by diluting the solution volume to fourfold, and Gel‐MA solution was dialyzed against deionized water with MWCO = 12k–14k Da at 50 °C for 3 d. After that, the solution was lyophilized, and the product of white foam was stored at −20 °C for future using. The lyophilized Gel‐MA without further UV crosslinking was used as sealant control group.

### Synthesis of α‐Thiol,ω‐carboxyl telechelic polystyrene

Styrene was first purified with an Al_2_O_3_ alkaline base by column chromatography. Purified 2.0 g styrene and 0.182 g RAFT‐COOH (weight ratio of styrene/RAFT‐COOH = 4000/364) were added to the reactor and stirred at 132 °C for 6 h under nitrogen atmosphere. After the reaction, the reactor was cooled with cold water. Then 10 mL dimethylformamide was added and stirred until all adhesion products were dissolved, then the solution was transferred in 30 mL methanol, and sediments were separated and collected. The collected products were air‐dried overnight. Dried products were added in 10 mL toluene and stirred until fully dissolved in a nitrogen atmosphere. 1 mL of 1‐butylamine was added dropwise and stirred for another 30 min; the color of the solution changed to light yellow. After the reaction, the solution was transferred in 40 mL methanol and stored at −4 °C for 4 h. The methanol was then removed by rotary evaporating at 40 °C. 20 mL toluene was then added to methanol‐free products and stirred for 30 min; the solution was mixed with 40 mL hexanes and stored at room temperature overnight. The final solid was crystallized from hexanes to obtain white solid (PS) and stored at room temperature for further use.

### Preparation of Gelatin Methacryloyl‐Polystyrene

The Gel‐MA‐P was prepared by fully dissolving a certain amount of Gel‐MA and HS‐PS‐COOH, and 1‐butylamine in 12 mL DMSO and stirred at 40 °C overnight. After that, the solution was added into 30 mL ether first, then transferred into 30 mL tetrahydrofuran and stirred for 2 h, and washed the solid products with deionized water three times. After that, the solution was lyophilized, and the product was stored at −4 °C for further use. The Gel‐MA‐Ps with various proportions (Gel‐MA‐0.5P, Gel‐MA‐P, and Gel‐MA‐2P) were prepared with different ingredient proportions by weight ratio of Gel‐MA/PS; also, the mass ratio of PS/1‐butylamine keeps at 10/1. For Gel‐MA‐0.5P, the mass ratio of Gel‐MA/P = 1/0.5; Gel‐MA‐P is Gel‐MA/PS = 1/1, and Gel‐MA‐2P is Gel‐MA/PS = 1/2 in mass ratio, respectively. For example, to prepare the group of Gel‐MA‐0.5PS, 600 mg Gel‐MA, 300 mg PS‐COOH, and 30 mg 1‐butylamine were dissolved in 12 mL DMSO and stirred at 40 °C overnight. Before using, 60 mg Gel‐MA‐P was fully dissolved in 2 mL DMSO/dH_2_O (30 mg mL^−1^), then transferred into petri‐dish (60 mm diameter) and air‐dried at room temperature overnight. Finally, a thin Gel‐MA‐P film was obtained and ready to use.

### Nuclear Magnetic Resonance (NMR) Analysis

^1^H NMR spectra of Gel‐MA, Gel‐MA‐0.5P, Gel‐MA‐P, and Gel‐MA‐2P samples were characterized by an Advanced 300 spectrometer. Samples were dissolved in dimethyl sulfoxide‐d_6_ (DMSO‐d_6_) with a concentration of 10 mg mL^−1^ (1 w/v%), respectively.

### FTIR Analysis

A Nicolet iS10 FTIR spectrometer was used to evaluate the characteristics among gelatin, Gel‐MA, and Gel‐MA‐2P, and 32 scan per sample with a resolution of 4. Purchased gelatin powder, lyophilized Gel‐MA, and prepared Gel‐MA‐2P film were used to record ATR‐FTIR spectra.

### Water Contact Angle Measurement

The water contact angles were measured in triplicate with a goniometer (Sheng Ding, China) on different membranes (Gel‐MA, Gel‐MA‐0.5P, Gel‐MA‐P, and Gel‐MA‐2P). A 5 µL distilled water droplet was dropped on the membrane surface; a CCD camera of the contact angle setup was used to capture the images, and the static contact angles were measured within 5 s.^[^ ^53^ ^]^

### SEM

PRP was added to 2.0 mg of each material and incubate at 37 °C for 1 h. They were then fixed with 2.5% of glutaraldehyde for 2 h, followed by dehydration in graded ethanol and drying. SEM imaging (Zeiss Crossbeam 340) was performed after samples were sprayed with gold.

### Platelet Adhesion Calculation

After PRP was incubated different sealants as described in SEM procedure above, the quantification of platelet adhesion was performed through the measurement of LDH released upon lysis of the adherent platelets with a Triton buffer. A calibration curve was generated by counting a known number of platelets using a microplate reader (Thermo varioskan flash). From the calibration curve, the platelet adhesion on sealants was determined.

### Water Absorption Test

The ability of water absorption of sealants was examined by incubating different kinds of sealants with PRP at 37 °C for 2 min. Furthermore, these immersed sealants would be collected to remove excessive liquid with filter paper to obtain the wet weight. They were then lyophilized and weighed to get the dry weight. Therefore, the water absorption rate could be calculated as: Water absorption (%) = [(Wet weight) − (Dry weight)]/(Dry weight) × 100%.

### LDH Release Assay

Cytotoxicity of the sealants was determined by LDH assay. NSCs were cultured with 2 mg of Gel‐MA, Gel‐MA‐0.5P, Gel‐MA‐P, and Gel‐MA‐2P in a 24‐well plate, respectively, for 24 h. Together with the control group without sealant, the supernatant was collected in a 96‐well plate to incubate with LDH working solution for 30 min in dark. Absorbance of LDH release was further tested at the wavelength of 490 nm with a microplate reader (Thermo varioskan flash). Value of NSCs lysed with 2.5% of Triton X‐100 was as the maximal release.

### Hemolysis Ratio

All sealants were put into centrifuge tubes containing 1.0 mL of saline each. Positive and negative controls were also prepared using equal amounts of ultrapure water and saline without any sample. Whole blood (0.2 mL) containing trisodium citrate dihydrate was diluted with saline (2.5 mL), and 200 µL of the diluted blood was added to each tube. The tubes were then incubated at 37 °C for 1 h. Finally, all blood samples were centrifuged at 1500 *g* for 5 min to measure the amount of hemoglobin released with a microplate reader (Thermo varioskan flash) at 540 nm and calculated with the equation:

(1) $$\mathrm{Hemolysis}\;\mathrm{ratio}\;\;\left(\%\right)=\frac{A_{\mathrm{sample}}\;-\;A_{\mathrm{negative}\;\mathrm{control}}}{A_{\mathrm{positive}\;\mathrm{control}}\;-\;A_{\mathrm{negative}\;\mathrm{control}}}\;\times 100$$

### TBI Model and Sealants Implantation

A controlled cortical impact (CCI) device set on a stereotaxic apparatus was applied to perform the TBI model as described.^[^ ^54^ ^]^ All mice were anesthetized using 2.5% isoflurane until no reaction was to the tail pinch test. Maintained anesthesia was conducted with 1.5% isoflurane by fixing mice on the stereotaxic apparatus with a gas mask. Craniectomy was performed to create a skull window of 3.0 mm × 4.0 mm on right parietal cortex, followed by CCI (3.0 mm lateral to midline and 1.0 mm anterior to bregma) with cylinder dropping on the impact tip (velocity: 4.0 m s^−1^, depth: 2.5 mm). The mice in sham group received the craniectomy only. After the injury, sealants of 5.0 mg from each group were inserted into the lesion sites, and incision was then sutured. Simultaneously, hemostatic time was recorded. Animals were placed in a heating chamber under 37 °C to be closely monitored until recovery from anesthesia. Mice which died during or immediately after the surgery would be counted and excluded.

### In Vivo Fluorescence Imaging

On 0, 1, 2, and 3 d postoperation, mice in control, Gel‐MA (AF488), and Gel‐MA (AF488)−2P groups were imaged with an in vivo imaging system (Fusion FX. Edge). Animals were anesthetized with 5% chloral hydrate (100 µg g^−1^ per body weight) intraperitoneally. After mice were positioned in the container, scanning protocol with an excitation wavelength of 488 nm was operated.

### Magnetic Resonance Imaging

Mice in control, Gel‐MA, and Gel‐MA‐2P groups underwent MRI (7.0T scanner, Bruker, USA) on 1 d postoperation. Animals were anesthetized with 2.5% isoflurane as induction and 1.5% isoflurane during the imaging. The scanning protocol applied T2 sequence on all mice, and 28 coronal slices were obtained per animal with the thickness of 0.5 mm. Impact area and inserted sealants were exhibited.

### Brain Water Content

At the time point 1, 3, and 7 d, brain water content of mice was calculated to assess the brain edema. As discussed,^[^ ^55^ ^]^ brains were removed from mice under anesthesia with injection of 5% chloral hydrate intraperitoneally and weighed rapidly to obtain the wet weight. They were then placed into an oven at 80 °C for 48 h and weighed to get the dry weight. Water content (%) = [(Wet weight) − (Dry weight)]/(Wet weight) × 100%.

### Garcia's Neurologic Evaluation

Animals’ neurologic functions were evaluated with Garcia's Neuroscore as described.^[^ ^56^ ^]^ To be specific, spontaneous activity (3 min), side stroking, vibrissae touch, limb extension, forepaw outstretching and climbing (1 min) of mice were evaluated from score 0 to 3, so this scale system ranges from 0 (maximal deficit) to 18 (intact). Mice were evaluated everyday postoperation with the scoring system from 1 to 7 d.

### TUNEL Assay

Animals from different time points were anesthetized with injection of 5% chloral hydrate intraperitoneally and perfused with saline and 4% of paraformaldehyde. Brains were removed to obtain 30‐µm‐thick slices via cryosection. TdT buffer, fluorescence labeling buffer, and TUNEL reaction buffer were prepared according to the manufacturer. Brain slices were washed with PBS for 10 min × 2 times, followed by PBST (PBS containing 0.5% of triton X‐100) permeabilization. The buffers above were mixed and 50 µL was added to each sample to incubate for 1 h in dark. Three washes with PBS and DAPI staining were then followed.

### Quantitative Real‐Time Polymerase Chain Reaction

Total RNA of brain samples was extracted with the RNA Extraction Kit. cDNA synthesis was then performed by RNA reverse transcription. According to manufacturer's protocol, qPCR was performed with the SYBR Premix Ex Taq II in CFX96 System (Bio‐Rad, USA). Expression normalization was processed against GAPDH and analyzed with the threshold (*F* = 2^−ΔΔCt^). Primer sequences are listed in **Table** **1**.

**Table 1:** Sequences of primers used in qPCR

	Forward	Reverse
Mbp	5′‐TCACAGCGATCCAAGTACCTG‐3′	5′‐CCCCTGTCACCGCTAAAGAA‐3′
Olig2	5′‐GGCGGTGGCTTCAAGTCAT‐3′	5′‐CATGGCGATGTTGAGGTCG‐3′
Tubb3	5′‐CCCAGCGGCAACTATGTAGG‐3′	5′‐CCAGACCGAACACTGTCCA‐3′
Rbfox3	5′‐GTAGAGGGACGGAAAATTGAGG‐3′	5′‐GTGGGGTAGGGGAAACTGG‐3′
Gfap	5′‐CGGAGACGCATCACCTCTG‐3′	5′‐TGGAGGAGTCATTCGAGACAA‐3′
Gapdh	5′‐AGGTCGGTGTGAACGGATTTG‐3′	5′‐GGGGTCGTTGATGGCAACA‐3′

### Western Blots

Platelet‐rich plasma was mixed with 2.0 mg of materials and incubated at 37 °C for 1 h. RIPA lysis with protease inhibitor cocktail was added to gently shake for 20 min. A BCA kit was used to examine the protein concentration. Following the loading buffer mix and boiling, 20 µg of protein samples was loaded on sodium dodecyl sulfate‐polyacrylamide gel electrophoresis (SDS‐PAGE) gel to conduct the immunoblotting. Primary antibodies applied were as follow: FTH (rabbit, 1:1000), FTL (rabbit, 1:1000), FBXL5 (rabbit, 1:1000), IRP2 (rabbit, 1:500), PI3K (rabbit, 1:1000), PKC‐α (rabbit, 1:1000), and GAPDH (mouse, 1:500). Secondary antibodies: HRP‐conjugated goat anti‐rabbit (1:10 000) and HRP‐conjugated goat anti‐mouse (1:10 000). Semiquantification was processed with QuantityOne software (Bio‐Rad).

### Immunofluorescence Staining

Brain slices from sham, control, and treatment groups were prepared by cryosection as introduced above. After PBST permeabilization, 1% of BSA prepared in TBST was added for blocking for 1 h at room temperature. Then primary antibodies diluted in primary antibody dilution buffer were added to slices for incubation under 4 °C overnight. On the next day, slices were washed three times with PBS and incubated with secondary antibodies for 1 h in dark at room temperature. Following three washes with PBS, DAPI was applied to label the nuclei. The primary antibodies were as below: MBP (rabbit, 1:200) and NeuN (mouse, 1:200). Secondary antibodies were as follows: Alexa‐488 conjugated goat anti‐rabbit (1:500) and Alexa‐594 conjugated goat anti‐mouse (1:500). Images of ten random fields on a slice were snapped with a fluorescent microscope (Zeiss) and positive rates of each were quantified systematically.

### Statistics

Means ± standard error of the mean was applied to express the values. The error bars were expressed as the standard error of means. One‐way analysis of variance (ANOVA) was used to compare values from different groups in the study. The difference was considered to be significant if *p*‐value < 0.05.

## Conflict of Interest

The authors declare no conflict of interest.

## Supporting information

Supporting Information

Supplemental Movie 1

Supplemental Movie 2

Supplemental Movie 3

## Data Availability

The data that support the findings of this study are available from the corresponding author upon reasonable request.
