# Supplementary material for: Hydrophobic Polystyrene‐Modified Gelatin Enhances Fast Hemostasis and Tissue Regeneration in Traumatic Brain Injury
Source: Adv Healthc Mater. 2023 Jul 23;12(30):2300708. doi: 10.1002/adhm.202300708 (PMC11468692; doi:10.1002/adhm.202300708)
Supplement: Supplementary file 1 — Supporting Information [file ADHM-12-2300708-s001.pdf]

ADVANCED HEALTHCARE MATERIALS

Supporting Information

for *Adv. Healthcare Mater.*, DOI 10.1002/adhm.202300708

Hydrophobic Polystyrene-Modified Gelatin Enhances Fast Hemostasis and Tissue Regeneration in Traumatic Brain Injury

Wenyan Li, Kaige Xu, Yuqing Liu, Xuejiao Lei, Xufang Ru, Peiwen Guo, Hua Feng, Yujie Chen* and Malcolm Xing**

Supporting Information

Hydrophobic polystyrene-modified gelatin enhances fast hemostasis and tissue regeneration in traumatic brain injury

Wenyan Li, Kaige Xu, Yuqing Liu, Xuejiao Lei, Xufang Ru, Peiwen Guo, Hua Feng, Yujie Chen*, Malcolm Xing**

W. Li, X. Lei, X. Ru, P. Guo, H. Feng, Y. Chen

Department of Neurosurgery, Southwest Hospital, Third Military Medical University (Army Medical University), Chongqing 400038, China

Email: fenghua8888@vip.163.com; yujiechen6886@foxmail.com

K. Xu, Y. Liu, M. Xing

Department of Mechanical Engineering, University of Manitoba, 75 Chancellors Circle, Winnipeg, MB R3T 5V6, Canada

Email: malcolm.xing@umanitoba.ca

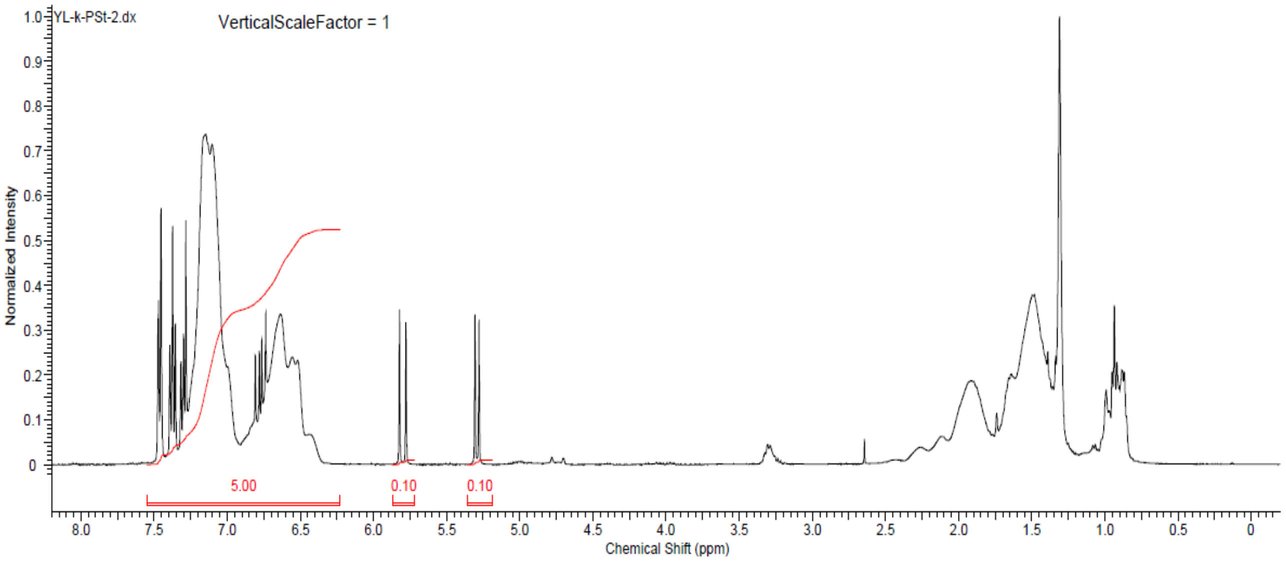

Figure S1. ^{1}H NMR spectrum of the polystyrene.

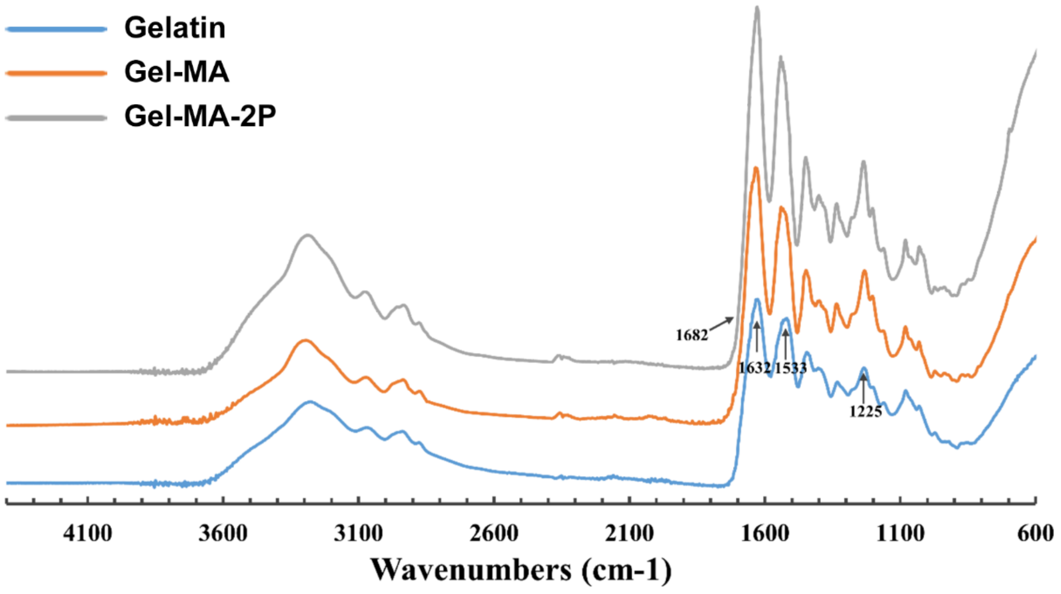

Figure S2. FTIR spectra of Gel-MA-2P, Gel-MA, and gelatin.

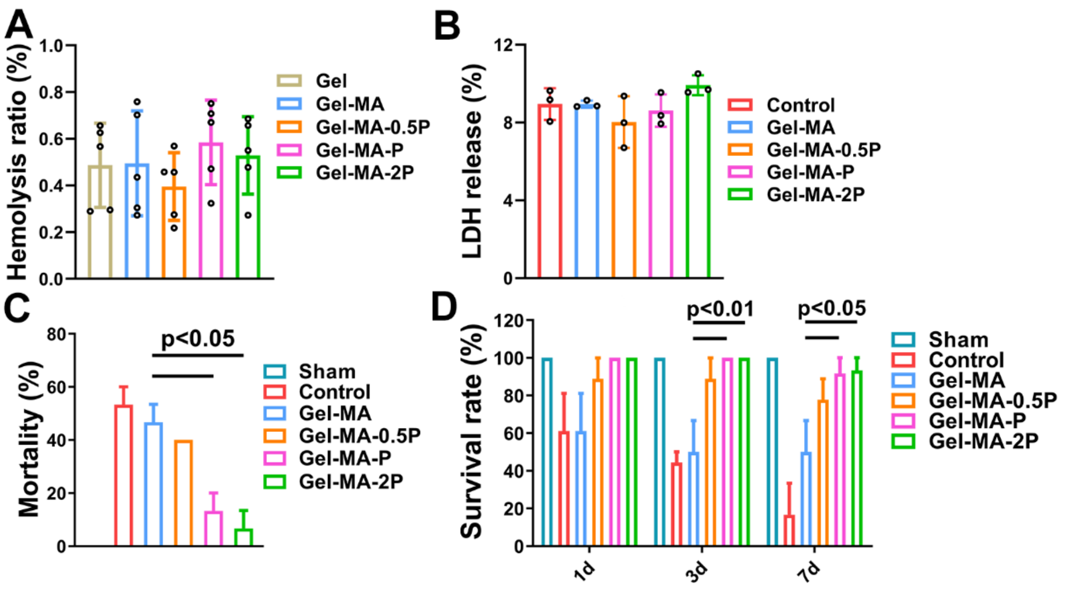

Figure S3. Hemocompatibility and cytotoxicity of sealants *in vitro* and *in vivo*. A) Hemolysis ratio of different sealants after incubation with whole blood. (n=5). B) Neural stem cells (NSCs) were cultured with or without sealants and then the supernatant was collected to process the lactate dehydrogenase (LDH) release assay to examine the biocompatibility of materials. (n=3). C) Mice mortality rates in surgery or immediately after surgery were calculated. (n=5). D) On day 1, day 3 and day 7, survival of mice in different groups was analyzed. (n=5).

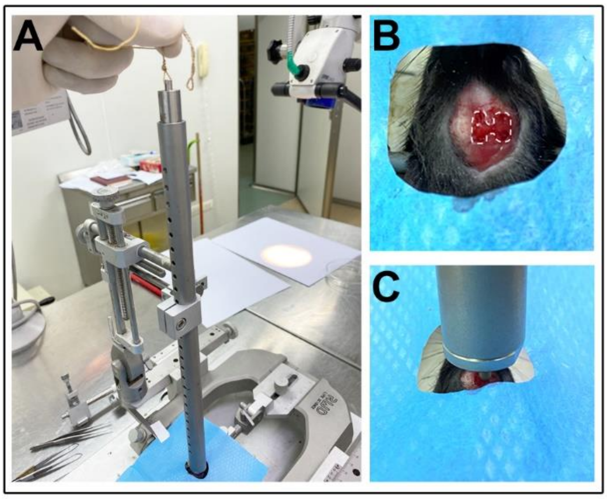

Figure S4. A) The cortical impact device on a stereotaxic apparatus. B)-C) Skull window and the operational field on the animal.

Movie S1. Swelling of different sealants following incubation with platelet-rich plasma. **Movie S2**. Dynamic contact angle test for Gel-MA, Gel-MA-0.5P, Gel-MA-P and Gel-MA-2P.

Movie S3. Traumatic brain injury model and sealant implantation on animals applying Gel-MA and Gel-MA-2P.
